# Outpatient Non-operative Management of Uncomplicated Acute Appendicitis: A Non-inferiority Study

**DOI:** 10.1007/s00268-023-07065-7

**Published:** 2023-05-20

**Authors:** Marco Ceresoli, Chiara Fumagalli, Paola Fugazzola, Nicola Zanini, Stefano Magnone, Michela Ravasi, Jacopo Bonalumi, Daniele Morezzi, Raffaele Bova, Benedetta Sargenti, Luca Schiavone, Alessandro Lucianetti, Fausto Catena, Luca Ansaloni, Marco Braga

**Affiliations:** 1 General and Emergency Surgery, School of Medicine and Surgery, University of Milano-Bicocca, Via Pergolesi 33, 20900 Monza, Italy; 2 General and Emergency Surgery, Fondazione IRCCS San Matteo, Pavia, Italy; 3 General and Emergency Surgery, Bufalini Hospital, Cesena, Italy; 4 General and Emergency Surgery, ASST Papa Giovanni XXIII, Bergamo, Italy

## Abstract

**Introduction:**

Non-operative management (NOM) of uncomplicated acute appendicitis is a well-established alternative to upfront surgery. The administration of intravenous broad-spectrum antibiotics is usually performed in hospital, and only one study described outpatient NOM. The aim of this multicentre retrospective non-inferiority study was to evaluate both safety and non-inferiority of outpatient compared to inpatient NOM in uncomplicated acute appendicitis.

**Methods:**

The study included 668 consecutive patients with uncomplicated acute appendicitis. Patients were treated according to the surgeon’s preference: 364 upfront appendectomy, 157 inpatient NOM (inNOM), and 147 outpatient NOM (outNOM). The primary endpoint was the 30-day appendectomy rate, with a non-inferiority limit of 5%. Secondary endpoints were negative appendectomy rate, 30-day unplanned emergency department (ED) visits, and length of stay.

**Results:**

30-day appendectomies were 16 (10.9%) in the outNOM group and 23 (14.6%) in the inNOM group (*p* = 0.327). OutNOM was non-inferior to inNOM with a risk difference of—3.80% 97.5% CI (− 12.57; 4.97). No difference was found between inNOM and outNOM groups for the number of complicated appendicitis (3 vs. 5) and negative appendectomy (1 vs. 0). Twenty-six (17.7%) outNOM patients required an unplanned ED visit after a median of 1 (1–4) days. In the outNOM group, the mean cumulative in-hospital stay was 0.89 (1.94) days compared with 3.94 (2.17) days in the inNOM group (*p* < 0.001).

**Conclusions:**

Outpatient NOM was non-inferior to inpatient NOM with regard to the 30-day appendectomy rate, while a shorter hospital stay was found in the outNOM group. Further, studies are required to confirm these findings.

**Supplementary Information:**

The online version contains supplementary material available at 10.1007/s00268-023-07065-7.

## Introduction

The management of acute appendicitis is a very debated topic with increasing interest worldwide. Since the description by Andersson, a conservative approach with antibiotic therapy has been re-proposed for uncomplicated acute appendicitis [1, 2]. Several randomized trials and meta-analyses demonstrated that conservative treatment is a safe alternative to appendectomy in uncomplicated acute appendicitis with similar short-term outcomes [3–11]. In particular, antibiotics were proved non-inferior to appendectomy with similar success proportion at a 30-day follow-up and lower morbidity rate. Existing trials proposed conservative management as in-hospital treatment coupling medical observation and intravenous broad-spectrum antibiotics. The non-response to non-operative management (NOM) has been reported to be 8.5%, and hospitalization would ensure patient safety with continuous re-evaluation, allowing prompt urgent appendectomy in case of failure of conservative treatment [8].

The APPAC II study demonstrated the non-inferiority of oral in-hospital antibiotics compared with intravenous route, opening new frontiers with the possibility of outpatient treatment [12]. The first experience of outpatient treatment of uncomplicated acute appendicitis has been described in a recent subgroup analysis of patients randomized and included in the CODA Trial [13]. Patients received a first dose of intravenous antibiotics in the emergency department, and then, they were discharged home within 24 h from the diagnosis of acute appendicitis. To the best of our knowledge, no other data are available in literature about completely outpatient non-operative management.

We hypothesized that the 30-day appendectomy rate among patients treated nonoperatively who were managed as outpatients would be non-inferior to that of those who were hospitalized. The aim of the study was to evaluate the non-inferiority and the safety of outpatient non-operative management of uncomplicated acute appendicitis.

## Methods

This is a multicentre retrospective observational study, including consecutive patients of any age treated for acute appendicitis at four Italian hospitals starting from January 2020. All these four hospitals share an updated treatment protocol for diagnosis and treatment of acute appendicitis according to existing international guidelines [14, 15]. Diagnostic work-up was performed with clinical evaluation, laboratory tests, and clinical scores (Alvarado and AIR score), followed by imaging confirmation of the diagnosis in all patients (US or CT scan according to the treating surgeon/emergency physician preference). The diagnosis of acute appendicitis was defined by the presence of any suggestive symptom confirmed by laboratory tests and coupled with either radiological confirmation or acute inflammatory response (AIR) score > 4 [16]. Exclusion criteria were negative imaging, AIR score < 5, and pre-operative diagnosis of complicated acute appendicitis defined as the presence of peritoneal abscess, perforation, peritonitis, severe sepsis, or shock.

According to the international guidelines on treatment of acute appendicitis [14, 15], patients with a pre-operative diagnosis of uncomplicated acute appendicitis underwent three different treatments based on the surgeon’s preference: upfront appendectomy, inpatient NOM (inNOM), and outpatient NOM (outNOM). Patients were grouped by an intention to treat principle. Patients discharged home within 12 h from the arrival in the emergency department were grouped in the outNOM group and received only oral antibiotics (amoxicillin/clavulanate or ciprofloxacin) according to the international guidelines [15]; inNOM patients were given intravenous antibiotics during hospitalization followed by oral antibiotics at home.

In all patients demographics, signs and symptoms of appendicitis, AIR and Alvarado scores, and imaging findings were collected in a dedicated database. Follow-up was carried out by phone calls and searching for possible readmissions in any Italian hospitals. Unplanned emergency department (ED) visits and hospitalizations were recorded.

The primary endpoint of the study was the 30-day appendectomy rate. Secondary endpoints were negative appendectomy rate, unplanned medical evaluation within 30 days, cumulative in-hospital stay, and long-term appendectomy rate.

### Statistical analysis

The sample size of our study was calculated assuming the 30-day appendectomy rates in both NOM groups reported in the CODA trial as reference [13]. With a statistical power of 80% and alpha = 0.025 (one side test), 284 patients (142 per group) are required to demonstrate the non-inferiority of the outNOM treatment with a non-inferiority limit of 5%.

Continuous data are reported as median and interquartile range and mean with standard deviation, while categorical data are reported as number and proportion. Categorical variables have been compared by Chi-square test and continuous variables by Mann–Witney’s *U* test. Variables significantly associated with the treatment choice have been identified by multiple logistic regression model. Non-inferiority of outNOM was tested evaluating the upper limit of the 97.5% confidence interval of the 30-day appendectomy risk difference. Predictors of 30-day appendectomy were evaluated with a logistic regression analysis and shown as adjusted odd ratio along with 95% confidence intervals. Long-term risk for appendectomy has been evaluated by Kaplan–Meier method and compared with the log-rank test. Statistics were performed with SPSS 28 (IBM Corp. Released 2021. IBM SPSS Statistics for Windows, Version 28.0. Armonk, NY: IBM Corp).

## Results

**Fig. 1:**
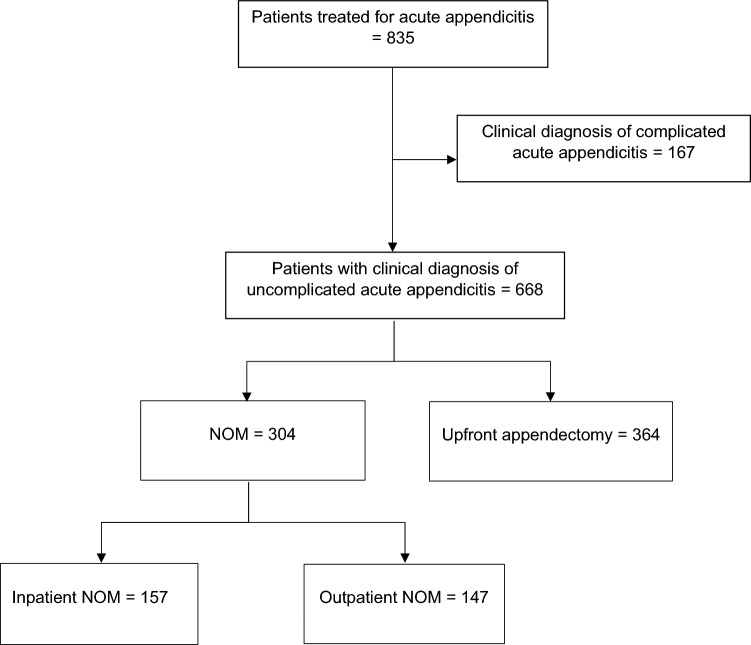
Study flow diagram

A total number of 668 patients with a diagnosis of uncomplicated acute appendicitis and fulfilling the inclusion criteria were retrieved between January 2020 and April 2022. Of them, 364 (54.3%) patients were given upfront appendectomy, 157 (23.5%) patients were admitted for inNOM, and 147 (22.1%) patients were discharged home for outNOM. Figure 1 shows the flow diagram of the study.

The characteristics of patients who were given upfront surgery or NOM are reported in the Supplemental Table 1. Multivariate analysis shows that male gender (OR 1.65), WBC count (OR 1.07), detection of appendicolith (OR 2.86), free fluid at US (OR 1.87), and CT confirmed diagnosis (OR 2.14) were associated to upfront appendectomy (Supplementary Table 2). Among the 364 patients who were given upfront surgery, the negative appendectomy rate was 8.8% (32 patients), while 33 (9.1%) had an intraoperative diagnosis of complicated appendicitis. Minor complications occurred in 18 (4.9%) patients. There was no major complications or post-operative mortality. Median length of hospital stay was 3 (2–5) days.

**Table 1:** Characteristics of patients treated with outpatient NOM and inpatient NOM

	Outpatient NOM (*n* = 147)	Inpatient NOM (*n* = 157)	*p* value
*Sex*			
Woman	72 (49.0)	74 (47.1)	0.853
Man	75 (51.0)	83 (52.9)	
Age*	27 (19–41)	37 (24–54)	< 0.001
Comorbidity	8 (5.40)	22 (14.0)	0.008
Cardiovascular	4 (2.70)	9 (5.7)	0.138
Respiratory	1 (0.70)	3 (1.9)	0.297
Liver	0 (0.00)	1 (0.6)	0.657
Kidney	0 (0.00)	1 (0.6)	0.846
Diabetes	1 (0.70)	4 (2.5)	0.164
HIV	0 (0.00)	4 (2.5)	0.04
Pregnancy	1 (0.70)	1 (0.6)	0.987
Cancer	1 (0.70)	4 (2.5)	0.164
Hours in ED*	5.65 (4.20–9.42)	9.9 (5.00–15.50)	< 0.001
Days from symptoms onset*	1 (1–2)	2 (1–2)	< 0.001
Temperature*	36.4 (36.00–37.40)	36.3 (36.00–37.70)	0.974
WBC (× 10^9/L)*	12.68 (10.36–14.60)	12.7 (10.10–15.60)	0.435
% Polymorphonuclear leukocytes*	77 (72–84)	82 (74–86)	0.233
CRP (mg/dl)*	2.72 (1.01–5.61)	3.7 (1.00–9.10)	0.236
Alvarado score*	6 (8–8)	6 (5–7)	0.085
AIR score*	5 (5–6)	5 (4–6)	0.208
US	130 (88.40)	124 (87.30)	0.636
Appendicolith	4 (2.70)	16 (10.2)	< 0.001
Appendix diameter (mm)*	9 (6–8)	10 (9–12)	< 0.001
Free fluid	22 (15.00)	58 (36.9)	< 0.001
CT scan	12 (8.20)	46 (29.3)	< 0.001
*Antibiotic therapy*			
Amoxi/clavulanate	49 (31.2)	128 (87)	
Ciprofloxacine/metronidazole	2 (1.3)	6 (4.1)	
Ertapenem	99 (63)	0	
Other	7 (4.5)	13 (8.9)	
Days of antibiotics*	2 (1–3) + 5 (5–7)	6 (5–7)	< 0.001

Values in parentheses are percentages unless indicated otherwise

*ED* emergency department, *WBC* white blood cells, *CRP* C reactive protein, *US* ultrasound, *CT* computed tomography

*Values are median and interquartile range (IQR)

Table 1 shows the characteristics of patients treated with NOM. Patients receiving inNOM had a higher detection of both appendicolith and free intraperitoneal fluid and a larger appendix diameter at US (*p* < 0.001). Patients receiving outNOM were younger (*p* < 0.001), had early onset of symptoms (*p* < 0.001) and lower comorbidity rate (*p* < 0.008). There was no significant difference between inNOM and outNOM groups for fever, white blood cells count, C-reactive protein level, and both Alvarado and AIR scores. Multiple regression analysis confirmed that age, presence of appendicolith, detection of intraperitoneal free fluid, and CT scan confirmed diagnosis were significantly associated with inNOM (Table 2). One pregnant patient was treated with InNOM and one with OutNOM: they were at first trimester pregnancy and conservative treatment was shared by surgeon and obstetrician team. Treatment was successful in both cases.

**Table 2:** Variables associated to outpatient NOM

	Univariate analysis		Multiple regression	
	OR	*p* value	OR	*p* value
Sex	0.957 (0.603–1.519)	0.853		
Age	0.969 (0.955–0.983)	< 0.001	0.980 (0.963–0.997)	0.019
Hours in ED	0.913 (0.868–0.959)	< 0.001		
Comorbidity	0.332 (0.142–0.776)	0.011	0.580 (0.221–1.526)	0.270
Cardiovascular	0.413 (0.124–1.374)	0.149		
Respiratory	0.317 (0.033–3.087)	0.323		
Diabetes	0.236 (0.026–2.140)	0.199		
Pregnancy	0.966 (0.060–15.590)	0.980		
Days from symptom onset	0.792 (0.678–0.925)	0.003		
Temperature	0.915 (0.708–1.182)	0.497		
WBC (× 10^)/L)	0.962 (0.907–1.021)	0.199		
% Polymorphonuclear leukocytes	0.988 (0.954–1.023)	0.489		
CRP (mg/dl)	0.964 (0.923–1.007)	0.102		
Alvarado score	1.149 (0.988–1.335)	0.071		
AIR score	1.153 (0.976–1.362)	0.094		
US	0.319 (0.174–0.584)	< 0.001		
Appendicolith	0.256 (0.082–0.797)	0.019	0.351 (0.101–1.222)	0.100
Appendix diameter (mm)	0.789 (0.704–0.884)	< 0.001	0.975 (0.926–1.026)	0.332
Free fluid	0.314 (0.178–0.554)	< 0.001	0.287 (0.155–0.529)	< 0.001
CT scan	0.227 (0.113–0.454)	< 0.001	0.250 (0.113–0.553)	0.001

Values in parentheses are 95 per cent confidence intervals

*ED* emergency department, *WBC* white blood cells, *CRP* C reactive protein, *US* ultrasound, *CT* computed tomography

Table 3 shows the 30-day outcomes in both NOM groups. OutNOM was non-inferior to inNOM with a 30-day appendectomy (risk difference − 3.80 (97.5%C.I − 12.57; 4.97)). Only one patient had a negative appendectomy. Seventeen (10.82%) inNOM patients needed an early appendectomy during the first hospitalization and six (3.82%) underwent appendectomy at a second hospitalization after a median of 7 (3–23) days from first diagnosis. Among the 26 (17.7%) outNOM patients who required an unplanned ED evaluation, 16 underwent appendectomy after a median time of 1 (1–4) day from first discharge, seven were discharged home and three continued antibiotics in hospital. No major post-operative morbidity or adverse events related to delayed appendectomies were observed in both NOM groups. When adjusting for age, appendix diameter, duration of symptoms, free fluid, and appendicolith OutNOM treatment did not result different for the 30-day appendectomy risk when compared to InNOM (Adj-OR 0.450 (0.170–1.194), *p* = 0.190) (Table 4).

**Table 3:** Thirty-day outcome

	Outpatient NOM (*n* = 147)	Inpatient NOM (*n* = 157)	*p* value
30-day appendectomy	16 (10.9)	23 (14.6)	0.327
Index admission appendectomy	0	17 (10.8)	
Unplanned ED visit	26 (17.7)	7 (4.4)	< 0.001
Appendectomy	16 (61.5)	6 (85.7)	
NOM	3 (11.5)	0	
Discharged home	7 (27)	1 (14.3)	
Laparoscopic appendectomy	15	22	0.98
Complicated appendicitis	5	3	0.142
Negative appendectomy	0	1	0.308
Post-operative morbidity	3	3	0.541
Clavien I	1	2	
Clavien IIa	2	1	
Days to appendectomy*	1 (1*–*4)	2 (1*–*3)	0.234
Cumulative days in hospital*	0 (0*–*0)	3 (3*–*4)	< 0.001
Cumulative days in hospital^§^	0.89 (1.94)	3.94 (2.17)	< 0.001

Values in parentheses are percentages unless indicated otherwise; values are *median and interquartile range (IQR) and ^§^Mean ± SD. ED: emergency department;

**Table 4:** Multiple regression analysis on predictors of 30-day appendectomy

	Multiple regression	
	Adjusted OR	*p *value
Out-NOM	0.450 (0.170–1.194)	0.109
Age	1.011 (0.985–1.038)	0.392
Comorbidity*	0.351 (0.042–2.945)	0.335
Days from symptoms onset	0.963 (0.755–1.228)	0.765
Appendicolith	0.637 (0.074–5.419)	0.680
Appendix diameter (mm)	1.061 (0.979–1.15)	0.147
Free fluid at imaging	1.136 (0.401–3.215)	0.809

^*^Comorbidity includes any comorbidity (cardiological, respiratory, diabetes, kidney, liver, and HIV)

**Fig. 2:**
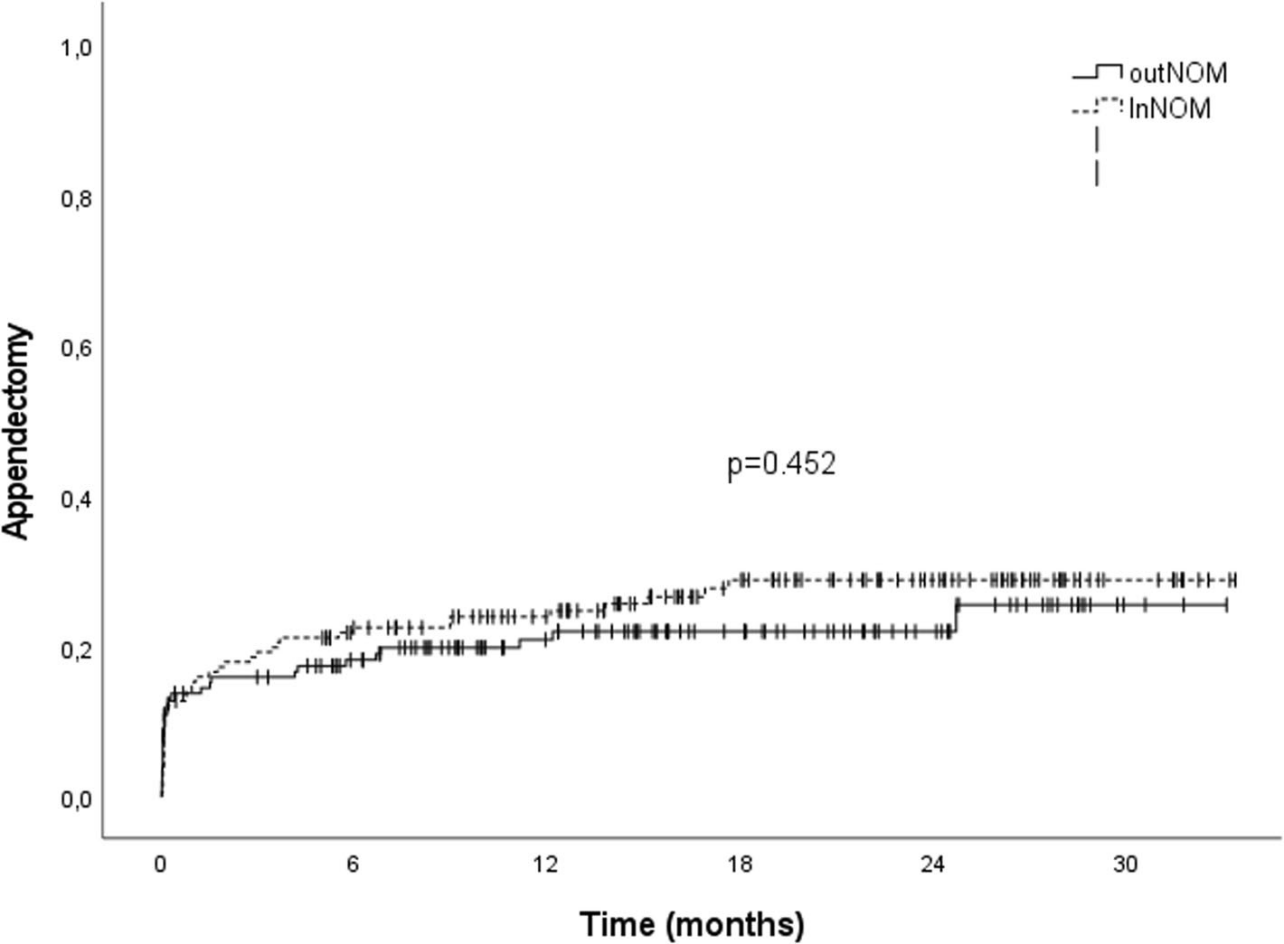
Long-term risk of appendectomy in both NOM groups

Overall long-term risk for appendectomy was 25% at 24 months without any difference between inNOM and outNOM groups (Fig. 2). No negative appendectomy was observed.

## Discussion

The results of the present study showed that outNOM was non-inferior to inNOM in patients with uncomplicated acute appendicitis. The 30-day appendectomy rate was similar in the two groups, while outNOM patients had shorter cumulative median hospital stay. In both NOM groups, the rate of patients operated for complicated acute appendicitis was below 4% and negative appendectomy rate was below 1%. At 30-day follow-up, a small proportion of patients required an unplanned emergency department visit for persistent or worsening symptoms.

In our series, about half of the patients with uncomplicated acute appendicitis were given upfront appendectomy according to surgeon’s preference. The majority of surgeons still consider NOM an ineffective treatment for uncomplicated appendicitis, even in the four participating hospitals. This is well reflected by two recent large international surveys reporting that NOM is the preferred treatment for only 14% of respondents [17, 18]. When compared to patients receiving NOM, patients who underwent upfront appendectomy had higher WBC count and AIR score, more frequent detection of appendicolith and free fluid at ultrasound/CT scan. All these variables might be considered as indicators of a more probable diagnosis of acute appendicitis. The presence of appendicolith is one of the stronger predictors of NOM failure which has an expected incidence up to 20% at 30 days [13]. Other known factors related to NOM failure are female gender and a larger appendix diameter [19].

The non-operative management of uncomplicated appendicitis was safe in our cohort of patients with short in-hospital stay, low morbidity, and recurrence rate similarly to the results obtained in large, randomized trials [3, 6]. Half of the patients were given out-hospital treatment with similar 30-day appendectomy rate when compared to inNOM patients. The results of our research were comparable to those reported by the CODA trial collaborative group [13], confirming that outpatient NOM is a valid option allowing a substantial reduction of unnecessary hospitalization and resources saving in patients with uncomplicated acute appendicitis. The meta-analysis published by Podda and coll showed that in-hospital NOM of uncomplicated acute appendicitis resulted in a pooled mean length of stay of 2.9 days with a mean cost of $ 2,509 [8].

A small proportion of outNOM patients required an early unplanned emergency department visit and subsequent appendectomy, as previously reported by the CODA trial. In our series, the vast majority of appendectomies were performed within one day after first discharge. A possible interpretation of this early failure might be the patient’s negative beliefs towards the out-hospital NOM success. The CODA trial showed a lower non-response rate in patients with positive beliefs when compared to patients with negative beliefs [20]. Obviously, outNOM patients have to accept the risk of possible disease recurrence and hospital readmission. Despite outNOM patients required more frequently an unplanned ED visit, the cumulative length of stay remained significantly lower in the OutNOM group when compared to the InNOM group.

The preoperative detection of appendicolith and free fluid are considered relative contraindications to NOM in uncomplicated acute appendicitis [15]. In our study, the majority of patients with pre-operative detections of free fluid and appendicolith were treated with upfront surgery according to the international guidelines [15]. A smaller proportion of these patients were proposed and accepted NOM. Interestingly, only 8.2% of patients with free fluid and 5.6% of patients with appendicolith underwent appendectomy within 30 days. At the multiple regression analysis, the presence of appendicolith and free fluid was not correlated with a higher 30-day appendectomy rate.

InNOM patients were older and had longer duration of symptoms, larger appendix diameter and higher proportion of appendicolith and free fluid. However, after adjusting for these variables, the 30-day appendectomy rate remained similar between the two groups.

The vast majority of outNOM patients were discharged with 6 day only oral amoxicillin/clavulanate therapy. The efficacy of non-operative management in our study was consistent with the results of CODA trial whose patients were given a combination of intravenous and oral antibiotics. Recently, the real need for antibiotic therapy in patients with uncomplicated appendicitis have been questioned by two randomized trials showing a similar non-response rate comparing NOM with or without antibiotics [21, 22].

This is a multicentre retrospective cohort study with a possible selection bias as main limitation. The patients’ allocation towards upfront surgery or NOM groups was guided by surgeon’s preference, reflecting the real clinical practice. In this complex scenario, it should also be taken into consideration, the effect of pandemic in changing the surgeon’s attitude to operative treatment of acute appendicitis [23]. Patients who were given outNOM had a significantly lower pre-operative detection of both appendicolith and intraperitoneal fluid, suggesting some objective criteria to address the out-hospital management. The large number of patients treated in a short-time period in four high-volume hospitals might be considered a strength of the present study as well as the nationwide long-term follow-up.

In conclusion, the present study shows that outpatient NOM is a non-inferior treatment when compared to inpatient NOM with regard to the 30-day appendectomy rate. Moreover, a shorter cumulative hospital stay was found in the outNOM group. Further large, randomized studies are needed to confirm the role of out-hospital NOM in uncomplicated appendicitis.

## Data availability statement

Data are available under request to the corresponding author, Dr. Marco Ceresoli (marco.ceresoli@unimib.it).

## Supplementary Information

Below is the link to the electronic supplementary material.

Supplementary file1 (DOCX 18 KB)

Supplementary file2 (DOCX 19 KB)

## Declarations

### Study preregistration

The study was not pre-registered in an independent registry.
