# Supplementary material for: Outpatient Non-operative Management of Uncomplicated Acute Appendicitis: A Non-inferiority Study
Source: World J Surg. 2023 May 20;47(10):2378–85. doi: 10.1007/s00268-023-07065-7 (PMC10474178; doi:10.1007/s00268-023-07065-7)
Supplement: Supplementary file 1 — Supplementary file1 (DOCX 18 KB) [file 268_2023_7065_MOESM1_ESM.docx]

**Supplementary Table 1.** Patients characteristics

|  | |  |  |  |
| --- | --- | --- | --- | --- |
|  |  | Non-operative management  (n=304) | Upfront appendectomy  (n=364) | p value |
| Sex | Woman | 146 (48.0) | 132 (36.3) | 0.002 |
|  | Man | 158 (52.0) | 232 (63.7) |  |
| Age* | | 31 (22-48) | 29 (18-50) | 0.118 |
| Comorbidity |  | 30 (9.9) | 31 (8.5) | 0.479 |
| Cardiovascular |  | 13 (4.3) | 13 (3.6) | 0.479 |
| Respiratory |  | 4 (1.2) | 4 (1.1) | 0.699 |
| Liver |  | 1 (0.3) | 3 (0.8) | 0.08 |
| Kidney |  | 1 (0.3) | 1 (0.3) | 0.847 |
| Diabetes |  | 5 (1.6) | 5 (1.4) | 0.665 |
| HIV |  | 4 (1.3) | 1 (0.4) | 0.096 |
| Pregnancy |  | 2 (0.7) | 0 (0) | 0.105 |
| Cancer |  | 5 (1.6) | 9 (2.5) | 0.564 |
| Hours in ED* | | 8.02 (4.67 - 14.47) | 8.1 (5.12 - 12.26) | 0.342 |
| Days from symptoms onset* | | 1 (1-2) | 1 (1-2) | 0.348 |
| Temperature* | | 36.3 (36.00 - 37.50) | 36.4 (36.00 - 37.40) | 0.539 |
| WBC (x10^9/L)* | | 12.6 (10.30 - 15.30) | 14.2 (10.86 - 16.62) | <0.001 |
| % polymorphonuclear leukocytes* | | 79 (72 – 85) | 80 (74-85) | 0.272 |
| CRP (mg/dl)* | | 3.3 (1.00 - 6.70) | 3 (0.69 - 8.14) | 0.575 |
| Alvarado sco*re | | 6 (5-7) | 6 (5-7) | 0.825 |
| AIR score* | | 5 (5-6) | 6 (4-7) | 0.013 |
| US |  | 267 (87.8) | 303 (83.2) | 0.072 |
| Appendicolith |  | 20 (6.6) | 45 (12.4) | 0.008 |
| Appendix diameter (mm)* | | 9 (8-11) | 10 (8-12) | 0.267 |
| Free fluid |  | 80 (26.3) | 132 (36.3) | 0.002 |
| CT scan |  | 58 (19.1) | 129 (35.4) | <0.001 |

Values in parentheses are percentages unless indicated otherwise; * values are median and interquartile range (IQR). ED: emergency department; WBC: white blood cells; CRP: C reactive protein; US: ultrasound
