# Supplementary material for: Outpatient Non-operative Management of Uncomplicated Acute Appendicitis: A Non-inferiority Study
Source: World J Surg. 2023 May 20;47(10):2378–85. doi: 10.1007/s00268-023-07065-7 (PMC10474178; doi:10.1007/s00268-023-07065-7)
Supplement: Supplementary file 2 — Supplementary file2 (DOCX 19 KB) [file 268_2023_7065_MOESM2_ESM.docx]

**Supplementary table 2.** Variables associated to upfront appendectomy (upfront appendectomy compared to non-operative management)

|  | Univariate analysis | |  | Multiple regression | |
| --- | --- | --- | --- | --- | --- |
|  | OR | p value |  | OR | p value |
|  |  |  |  |  |  |
| Age | 0.992 (0.983-1.000) | 0.051 |  |  |  |
| Sex | 1.764 (1.278-2.435) | 0.001 |  | 1.656 (1.182-2.319) | 0.003 |
| Hours in ED | 0.988 (0.967-1.010) | 0.284 |  |  |  |
| Comorbidity | 0.751 (0.439-1.284) | 0.295 |  |  |  |
| Cardiovascular | 0.612 (0.278-1.347) | 0.223 |  |  |  |
| Respiratory | 0.531 (0.132-2.144) | 0.374 |  |  |  |
| Kidney | 0.535 (0.033-8.586) | 0.658 |  |  |  |
| Liver | 1.001 (0.999-1.002) | 0.999 |  |  |  |
| Diabetes | 0.53 (0.152-1.851) | 0.320 |  |  |  |
| HIV | 0.802 (0.133-4.834) | 0.810 |  |  |  |
| Pregnancy | 0.535 (0.033-8.586) | 0.658 |  |  |  |
| Cancer | 0.963 (0.319-2.909) | 0.947 |  |  |  |
| Days from symptoms onset | 0.948 (0.889-1.012) | 0.110 |  |  |  |
| Temperature | 0.996 (0.834-1.188) | 0.960 |  |  |  |
| WBC (x10^9/L) | 1.078 (1.038-1.120) | <0.001 |  | 1.070 (1.020-1.122) | 0.005 |
| % polymorphonuclear leukocytes | 0.997 (0.989-1.006) | 0.513 |  |  |  |
| CRP (mg/dl) | 1.014 (0.989-1.040) | 0.267 |  |  |  |
| Alvarado score | 1.032 (0.936-1.138) | 0.532 |  |  |  |
| AIR score | 1.122 (1.019-1.236) | 0.020 |  | 1.002 (0.889-1.130) | 0.973 |
| US | 1.436 (0.967-2.133) | 0.073 |  |  |  |
| Appendicolith | 2.131 (1.210-3.756) | 0.008 |  | 2.862 (1.466-5.587) | 0.002 |
| Appendix diameter | 1.007 (0.978-1.036) | 0.655 |  |  |  |
| Free fluid at US | 1.714 (4.43-2.402) | 0.002 |  | 1.874 (1.288-2.728) | 0.001 |
| CT scan | 2.522 (1.748-3.638) | <0.001 |  | 2.141 (1.441-3.181) | <0.001 |

Values in parentheses are 95 per cent confidence intervals. ED: emergency department; WBC: white blood cells; CRP: C reactive protein; US: ultrasound; CT computed tomography
